# Development of a novel multiplex electrochemiluminescent-based immunoassay to aid enterotoxigenic *Escherichia coli* vaccine development and evaluations

**DOI:** 10.1016/j.jim.2019.04.003

**Published:** 2019-07

**Authors:** Subhra Chakraborty, Jessica Brubaker, Clayton Harro, Thomas Weirzba, David Sack

**Affiliations:** a Department of International Health, Johns Hopkins Bloomberg School of Public Health, Baltimore, MD, USA; b PATH, Washington, DC, USA

**Keywords:** ETEC, Immune responses, Vaccine, Multiplex ECL, ELISA

## Abstract

Enterotoxigenic *Escherichia coli* (ETEC) is a leading cause of bacterial diarrhea both among children in low and middle income countries and in travelers to these regions. Although there are several approaches to develop an effective vaccine for ETEC, no licensed vaccines are currently available. The most advanced ETEC vaccine candidates include multiple colonization factors along with the heat labile toxin B subunit. In the absence of known correlates of protection, and to understand the mechanism of protection, monitoring immune responses to a majority of the vaccine associated antigens using various types of samples is needed. Unfortunately, a traditional ELISA is time consuming, labor intensive and requires substantial amounts of antigens and sample volumes. To address these constraints, we developed and validated a novel high throughput electrochemiluminescent (ECL) - based multiplex immunoassay using Meso Scale Discovery (MSD) platform for analyzing immune responses to ETEC antigens. The ETEC multiplex ECL assay is an 8-plex assay which includes the ETEC colonization factor antigens (CFA/I, CS1, CS2, CS3, CS5 and CS6) along with the two subunits of heat labile toxin (LTA and LTB). Our data suggested that a single dilution of sample provides a quantifiable result for a wide range of sample titers. To compare ETEC multiplex ECL with ELISA, we carried out assays using the same antigens with the two immunoassay platforms using a common sample set of serum and ALS (antibodies in lymphocyte supernatant) specimens. The MSD platform achieved excellent correlations with ELISA for the antigens tested, consistently detecting comparable antibody levels in the samples. The ETEC multiplex ECL can serve as a fundamental platform in evaluating performances of candidate ETEC vaccines in future field trials.

## Highlights

• A novel ETEC multiplex ECL assay was developed which achieved excellent correlations with conventional ELISA.
• ETEC multiplex ECL assay would minimize volumes of samples and antigens as well as save time and labor.
• The ETEC multiplex assay could serve as an improved platform for evaluation of candidate ETEC vaccines.

## 1 Introduction

Enterotoxigenic *Escherichia coli* (ETEC) is a leading bacterial cause of morbidity and mortality due to diarrhea in children in resource-poor settings (Qadri et al., 2005), (Bourgeois et al., 2016), (Liu et al., 2016), (Chakraborty et al., 2018). Studies have shown that children infected with ETEC are at higher risk of becoming stunted (Lozano et al., 2010; Murray et al., 2010; Vos et al., 2010), (Lamberti et al., 2014), (Bourgeois et al., 2016). ETEC are also the most frequent causes of diarrhea in the travelers and deploying military service members (Jiang et al., 2002), (Steffen and Connor, 2005), (Sack et al., 2007), (Hameed et al., 2016), (Rivera et al., 2013).

ETEC vaccine development has been a long standing WHO priority (Bourgeois et al., 2016). Although there are several approaches to develop an effective vaccine for ETEC, no licensed vaccines are currently available. A significant road block to successful vaccine development is our poor understanding of the antigens that elicit protective immune responses against ETEC illness and the immune mechanisms of protection (Chakraborty et al., 2015), Qadri et al., 2005), (Zhang and Sack, 2012).

ETEC are complex heterogenic pathogens. With many “O” serotypes and more than 26 colonization factors (CFs) so far identified (Del Canto et al., 2012), two enterotoxins present in different combinations, which makes the development of a broadly protective vaccine against ETEC very challenging. In the classical paradigm of ETEC pathogenesis, these organisms adhere to the small intestinal mucosa via CFs. Once intestinal colonization has occurred, ETEC strains elaborate heat-labile toxins (LT) and/or heat-stable toxins (ST) that disrupt fluid homeostasis, leading to fluid hyper-secretion and watery diarrhea (Qadri et al., 2005), (Chakraborty et al., 2015, Chakraborty et al., 2018). The majority of current ETEC vaccine candidates target the induction of anti-CF antibodies to block or interfere with colonization of the intestinal mucosa along with the induction of LT toxin neutralization antibodies. The most advanced ETEC vaccine candidates ACE527 and ETVAX (Harro et al., 2011), (Darsley et al., 2012), (Lundgren et al., 2014) includes the B subunit of the LT toxin along with 4 to 6 CF antigens (CFA/I, CS1, CS2, CS3, CS5 and CS6) which have been shown to be associated with ETEC strains causing clinical diarrhea in both travelers, as well as infants and young children living in the low and middle income countries. In addition to these conventional antigens, there are additional novel antigens which have shown to afford protection against ETEC infection in preclinical studies but are not included in the current ETEC vaccine candidates (Fleckenstein et al., 2013), (Luo et al., 2015).

The correlates of protection and or correlates of immunity of ETEC diarrhea are not yet known (Chakraborty et al., 2015). Therefore, to determine the immunogenicity of an ETEC vaccine and to understand the mechanism of protection, monitoring immune responses to a majority or all of these vaccine associated antigens using various systemic and mucosal samples is crucial. Measuring magnitudes and kinetics of immune responses using traditional enzyme linked immunosorbent assay (ELISA) is currently the mainstay of ETEC vaccine evaluation. In phase III vaccine trials and in the epidemiological studies of ETEC, evaluating the immune responses to these antigens by traditional ELISA will be extremely time consuming, labor intensive and more importantly would require substantive amount of antigens and sample volumes to fully assess the immunogenicity of candidate vaccines containing multiple CFs and toxoid antigens. Since the target age group for ETEC vaccines is children under 5 years, obtaining sufficient volumes of blood is a challenge.

To address this critical gap, here we have developed and validated a novel high throughput electrochemiluminescent (ECL) - based multiplex immunoassay using Meso Scale Discovery (MSD) platform for analyzing immune responses to ETEC antigens. This assay will allow testing 10 antigens per well in a 96 well plate. As a proof of concept, we developed an 8-plex assay which includes six ETEC colonization factor antigens and the two subunits of LT toxin. We assume our MSD-ETEC assay will optimize the use of the antigens and samples by significantly reducing antigen and sample volumes required by simultaneous multi-parameter assessments of immune responses to ETEC vaccine.

## 2 Materials and methods

### 2.1 Control samples

We have developed two standard samples of high and low titers by pooling serum from volunteers who were immunized with ACE527 ETEC vaccine and challenged with ETEC H10407 (Darsley et al., 2012). These samples were selected based on their titers in ELISA assay. We also used a pool of pre immune serum which was likely lacking antibodies against any of the test antigens. These control serum samples were used for all the optimization assays.

### 2.2 ACE527 ETEC vaccine

ACE527 is an oral, live attenuated, three-strain recombinant ETEC bacterial vaccine which includes the antigens CFA/I, CS1, CS2, CS3, CS5, CS6 and LTB (Harro et al., 2011; Darsley et al., 2012). The two-part phase IIb study conducted at Johns Hopkins University enrolled healthy American adults to receive ACE527 alone or a placebo. After demonstrating comparable safety and immunogenicity of both vaccine arms, volunteers were challenged with the wild type virulent strain ETEC H10407 (LT, STh, STp and CFA/I) (Darsley et al., 2012).

We used the serum and ALS (antibody in lymphocyte supernatant) samples from the volunteers vaccinated with ACE527 vaccine and placebo volunteers challenged with H10407 ETEC for validation of the multiplex ECL assay. Venous blood for serum and ALS were collected. For ALS, peripheral blood mononuclear cells (PBMCs) were isolated and incubated at 37 °C and 5% CO2 for 72 h with no antigenic stimulation as described previously (Chakraborty et al., 2015, Chakraborty et al., 2018). The supernatant fluid was cryopreserved and subsequently used in an ELISA and ECL to measure the concentration of antibody released by the PBMCs.

### 2.3 Antigens

The antigens used in this study were CFA/I, CS1, CS2, CS3, CS5, CS6; LTA and LTB subunits of enterotoxin LT. The antigens CFA/I, CS1, CS2, CS3 and CS6 were obtained from PATH; CS5 from Michael Prouty, Naval Medical Research Center, Bethesda, USA; LTA and LTB from John Clements, Tulane University School of Medicine, LA, USA; GM1 from Sigma (Sigma-Aldrich, St. Louis, MO).

### 2.4 Conventional ELISA

For the assessment of specific IgA titers, flat-bottom ELISA plates (Nunc, Roskilde, Denmark) were coated with purified antigens diluted in PBS. Samples were 3-folds serially diluted and tested in duplicates (Chakraborty et al., 2015, Chakraborty et al., 2018). A GM1-ELISA method was used for the determination of levels of LT-specific antibodies (Chakraborty et al., 2015, Chakraborty et al., 2018). Secondary antibodies used were goat anti-human IgG or IgA conjugated to horseradish peroxidase (HRP) (KPL, Gaithersburg, MD). For each assay, the endpoint titer was calculated as the reciprocal dilution giving rise to an absorbance value of 0.4 above the background at 450 nm.

### 2.5 Meso Scale Discovery platform and assay method

The Meso Scale Discovery (MSD) technology is based on ECL detection utilizing a Sulfo-tag label that emits light upon electrochemical stimulation. Using a dedicated ECL plate reader, an electrical current is placed across the plate-associated electrodes, resulting in a series of electrically induced reactions leading to a luminescent signal. In this system background signals are minimal because the stimulation mechanism (electricity) is decoupled from the signal (light). The multispot configuration used in development and validation was 10 spots/well in a 96-well plate format. Eight of the 10 spots were coated in this study. Each well was coated with specific concentration of the 8 antigens (33 μg/ml per spot unless specified otherwise for optimization studies). The control or test samples were diluted at appropriate dilutions in phosphate buffered saline (PBS) containing 1% Blocker A (BSA). Each antigen-coated plate was incubated at ambient temperature for 1 h on a shaker platform with 3% Blocker A (BSA) in PBS. Plates were washed with 0.05% PBS-Tween (PBS-T), and 25 μl per well of the diluted test samples were added and incubated for 2 h at ambient temperature on a shaker platform. Plates were washed with 0.05% PBS-T; a MSD Sulfo-tag-labeled-goat anti-human IgA or IgG secondary antibody was added to each well; and the mixture was incubated for 2 h at ambient temperature on a shaker platform. Plates were washed with 0.05% PBS-T, and 150 μl of MSD read buffer T (with surfactant) diluted 1:4 in water was added to each well. The plates were read using an MSD sector imager, model 2400. Data were then analyzed by the MSD workbench software provided by the manufacturer. The ECL plates were printed in the MSD printing facility at Meso Scale Discovery (Meso Scale Diagnostic LLC, Rockville, MD, USA).

### 2.6 Assay development approach

Based on the protocol for the conventional ELISA, (Harro et al., 2011), (Chakraborty et al., 2015, Chakraborty et al., 2018), (Darsley et al., 2012) assay parameters were optimized. The assays were first optimized with singleplex ECL-based immunoassay individually for 8 target antigens (Table 1). In the singleplex assays, antigen concentrations (1, 2 and 4 μg/ml), incubation time of serum samples (2 h and overnight) and 3 different concentrations of SULFO-TAG antibodies (0.5, 1 and 1.5 μg/ml) were evaluated. LT antigen assays with and without GM1 gangliosides were tested.

**Table 1:** Optimized parameters for ECL multiplex immunoassay.

Assay parameters	Tested conditions	Selected conditions
Antigens concentration (μg/ml)	33, 65, 130	33
Antigen coating reagents	PBS + stabilizer, PBS + BSA + stabilizer	PBS + stabilizer
Blocking reagent	3% blocker A (BSA)	3% blocker A (BSA)
Blocking time	1 h	1 h
Incubation time of samples (hours)	2 and 16 (overnight)	2
Sample dilutions for serum	1:10, 1:90, 1:810, 1:2430, 1:7290, 1:21870	1:1000
Secondary antibody concentrations	1, 2 and 4 μg/ml	1 μg/ml
Secondary antibody incubation (hours)	1, 2 and 16 (overnight)	2

The optimized parameters from the singleplex assays were transferred to multiplex platform and further validated using our standard samples. In addition, sample dilutions were optimized in the multiplex format. Parameters optimized in single and multiplex format assays are shown in Table 1. ECL plates were printed with 8 antigens CFA/I, CS1, CS2, CS3, CS5, CS6, LTA, LTB in each well of a 96 well plate in multiplex format. The antigens were selected based on the antigens that are included in the current candidate ETEC vaccines (Harro et al., 2011), (Darsley et al., 2012), (Lundgren et al., 2014). The signals were detected using the MSD sector imager 2400 settings. Similarly as serum, the assay conditions for ALS were optimized which includes antigen concentrations, dilution of samples, incubation times. The multiplex ECL results were then compared with conventional ELISA.

### 2.7 Multiplex ECL assay performance and reproducibility

Studies were carried out to analyze the reproducibility, precision and linearity of the assays.

#### 2.7.1 Reproducibility and precision

To establish the reproducibility and precision of the assay, we determined the total precision (intra- and inter- assay variability) of the assay. Intra-assay variability was determined as within-run variations (within the same plate) that represent the repeatability of the assay under the same conditions. Inter-assay variability was determined as how the mean precision varies with time between runs when the same specimens were assayed on different days.

#### 2.7.2 Dilution linearity

The high titer sample and a low titer serum sample were serially diluted to generate dilution curves. All the samples were run in the multiplex ECL plates.

#### 2.7.3 Specificity of the multiplex ECL assay

We measured the specificity of the assay using competitive inhibition assay. The high titer control serum was diluted 1:1000 in PBS + 1% blocker A (BSA). The serum was then divided into ten aliquots and each aliquot was incubated for one hour with 10 μg/ml of each antigen or serum diluent buffer only. In this assay, binding of the antigen with specific antibody in the serum inhibits the serum in further binding to that antigen coated on the plate. The percent inhibition was calculated using the formula [1 – (serum concentration in the presence of inhibitor/serum concentration in the absence of inhibitor)] × 100%.

### 2.8 Assay concordance experiments

The concordance between the multiplex ECL and ELISA assays was assessed using serum and ALS samples from volunteers who were immunized with ACE527 ETEC vaccine and challenged with ETEC H10407. Concordance among the two assays was assessed for all the 8 antigens.

### 2.9 Statistical methods

The percent coefficient of variations (CV) were used to evaluate the intra-assay and inter-assay precisions. The linearity was determined using coefficient of determination (R^2^). The comparison of ELISA and ETEC multiplex ECL were compared using a two sided *t*-test with 95% confidence interval. All graphs and statistical analysis were done with Graph- Pad Prism (version 5) software. Analysis was performed using the log transformed mean ECL units.

## 3 Results

### 3.1 Assay optimization

**Fig. 1:**
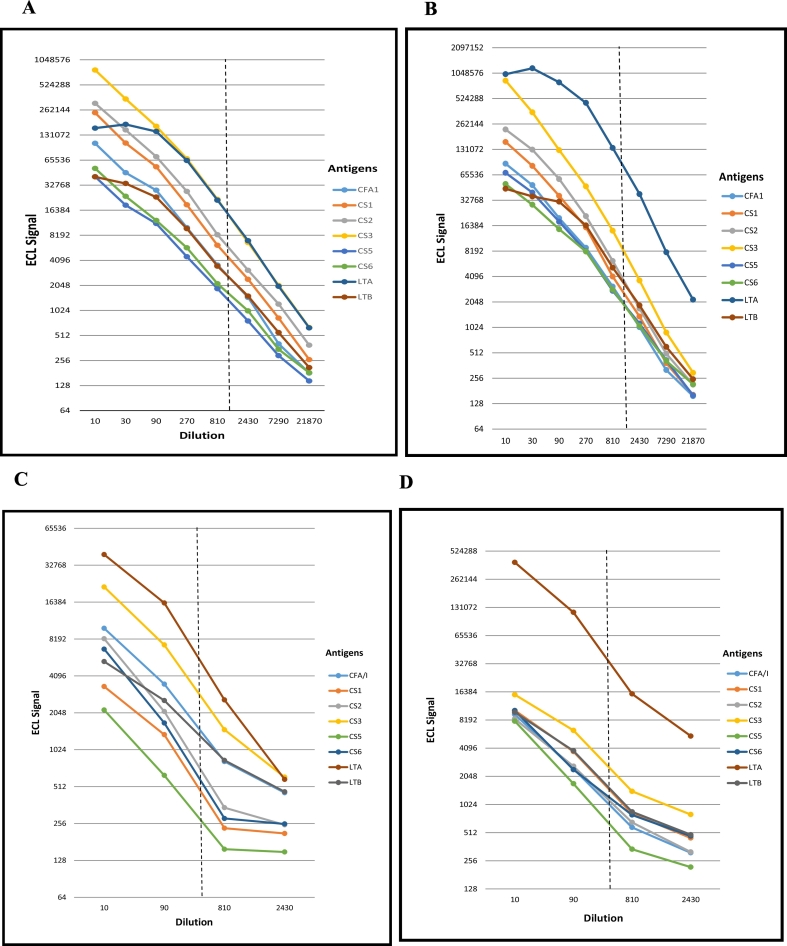
Linearity of high and low titer serum samples using ETEC multiplex ECL. A. high titer serum for IgA; B. high titer serum for IgG; C low titer serum for IgA; D low titer serum for IgG; dotted line indicates serum dilutions of 1:1000 for high titer samples and 1:500 for low titer sample.

The assay parameters for conventional and ECL singleplex immunoassays were optimized as listed in Table 1. Three different antigen concentrations 33 μg/ml, 65 μg/ml and 133 μg/ml were tested using low and high titer control serum and tested for all the 8 antigens (Fig. 1). We found similar performance between 33 μg/ml and 65 μg/ml concentrations except the signal intensity increased with the increase in antigen concentrations. However, with 130 μg/ml some antigens reached saturation. Since our objective was to find a concentration of antigens which was minimal as well as sufficient to detect the specific antibody titers, we selected 33 μg/ml as the optimal concentration for all the 8 antigens. Of the two blocking reagents tested, PBS + stabilizer (MSD proprietary) performed well however when BSA was added, the signals were lower. Hence, PBS + stabilizer were selected. In the ECL immunoassay, 1 μg/ml of the Sulfo-Tag antibody and incubation for 2 h were selected since these concentrations and incubation period provided the best signal/background ratio in all serum dilutions. Since there was no significant difference between the responses to LT antigen with and without GM1 gangliosides, the multiplex assays were performed without GM1.

### 3.2 Precision

Studies were carried out to analyze the performance and reproducibility of the assay using the established multiplex ECL assay conditions. To determine intra assay variability, control samples with high and low titers for IgA and IgG (dilution of 1:1000) were run for all 8 antigens on 5 different locations on a plate. The percent CV were below 20% (range 5.3%–18.1%) for all of the antigens (Table 2). Inter assay variability was evaluated by analyzing the data of responses to IgA and IgG of the 8 antigens using 10 individual serum samples (dilution of 1:1000) from the subjects immunized with ACE527 vaccine. Samples were run on 3 consecutive days by the same analyst on the same plate locations. The % CV for all the 8 antigens in the inter assay variability were below 20%, (range 7.7%–17.9%) (Table 3). The results from this analysis showed that there is no significant variations within a plate and among the days.

**Table 2:** Intra-assay variability: 2 samples high and low titers (dilution 1:1000) run in 5 locations on the plate.

Antigens	IgA		IgG	
	Sample with high titer % CV	Sample with low titer % CV	Sample with high titer % CV	Sample with low titer % CV
CFA/I	14.4	10.5	11.4	11.7
CS1	13.5	17.7	12.2	15.6
CS2	5.5	14.7	9.3	17.7
CS3	6.0	9.6	5.3	6.0
CS5	18.1	15.9	13.0	17.1
CS6	14.7	18.0	6.7	7.8
LTA	7.4	11.6	6.9	6.9
LTB	16.3	14.2	6.9	6.9

**Table 3:** Inter assay variability: ten serum samples run in duplicate in 3 consecutive days (dilution 1:1000).

Antigens	IgA	IgG
	Average % CV	Average % CV
CFA/I	8.7	12.6
CS1	9.1	11.8
CS2	14.5	11.3
CS3	10.8	10.7
CS5	10.5	15.6
CS6	11.7	13.1
LTA	7.7	12.6
LTB	17.9	16.2

### 3.3 Dilution linearity

The pool of high titer serum samples was diluted 3 folds serial dilutions (10 to 21,870 folds) to generate a full curve as shown in Fig. 1A (IgA) and 1B (IgG), along with a single point dilution (1:1000 dilution) of this pooled serum sample. The dilutions were tested for the IgA and IgG of all the 8 antigens. The coefficient of determination (R^2^) values were between 90 and 100% for both IgA and IgG (Table 4A). In addition, to understand the performance of low titer serum samples we performed similar assays using a very low titer serum sample with dilutions 1: 10, 90, 810 and 2430 [Fig. 1C (IgA), and 1D (IgG)]. The R^2^ values were between 75 and 90% for both IgA and IgG (Table 4B). When starting from a low ECL signal (Fig. 1C, D), at the dilutions higher than 810 the signals rich the lowest detection threshold. The R^2^ values improved when analyzed excluding the 2430 dilution (87% to 98%) (Table 4B). These data of high and low titer serum samples suggested that a single dilution 1:500 will provide a quantifiable result for a wide range of sample titers.

**Table 4A:** Dilution linearity (high titer).

Antigens	Coefficient of determination (R^2^)	
	IgA	IgG
CFA/I	0.9916	0.9935
CS1	0.9954	0.9911
CS2	0.9954	0.989
CS3	0.992	0.9943
CS5	0.996	0.9941
CS6	0.997	0.9907
LTA	0.9266	0.914
LTB	0.9707	0.945

**Table 4B:** Dilution linearity (Low titer).

Antigens	Coefficient of determination (R^2^)			
	IgA	IgA	IgG	IgG
	(All dilutions)	(Dilution 2430 excluded)	(All dilutions)	(Dilution 2430 excluded)
CFA/I	0.8378	0.946	0.8178	0.9015
CS1	0.8508	0.9747	0.8455	0.9535
CS2	0.7768	0.9068	0.8178	0.9249
CS3	0.8374	0.9434	0.8711	0.9729
CS5	0.7807	0.9156	0.7503	0.8795
CS6	0.7678	0.9036	0.7566	0.8733
LTA	0.8789	0.9749	0.8125	0.9305
LTB	0.9027	0.9827	0.857	0.9626

### 3.4 Specificity

**Fig. 2:**
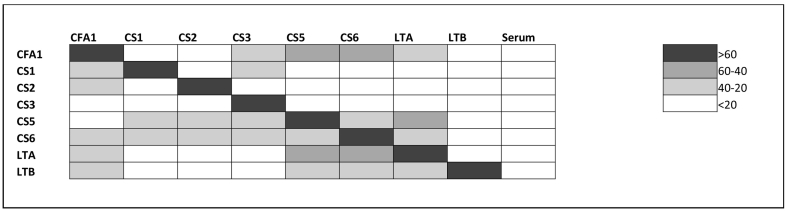
Specificity of ETEC multiplex ECL for 8 antigens using competitive inhibition assay.

We examined the effect of inhibition on binding of the high titer serum in a multiplex format. For all of the antigens the inhibition was over 60% (Fig. 2). There was some cross-reactivity observed between antigens. However, in each case the highest percent inhibition was observed with specific antigens, indicating specificity of the assay. The cross-reactivity observed may be due to similarity in the CF's structure and/or impurities leftover from the antigen purification process. This has also been found in conventional ELISA (Chakraborty et al. personal communication).

### 3.5 Performance of ETEC MSD using ALS samples

To test the performance of the multiplex ETEC ECL assay using a different matrix we used the ALS samples obtained from volunteers immunized with the ACE527 vaccine. We tested three concentrations of each of the 8 antigens and four sample dilutions (neat, 1:3, 1:9 and 1:81). We determined at the antigen concentration of 33 μg/ml per well in a 96 well plate the optimum sample dilution was 1:3. The ECL signals were very low or below detection limit using higher dilution of samples.

### 3.6 Multiplex ECL assay validation

**Fig. 3:**
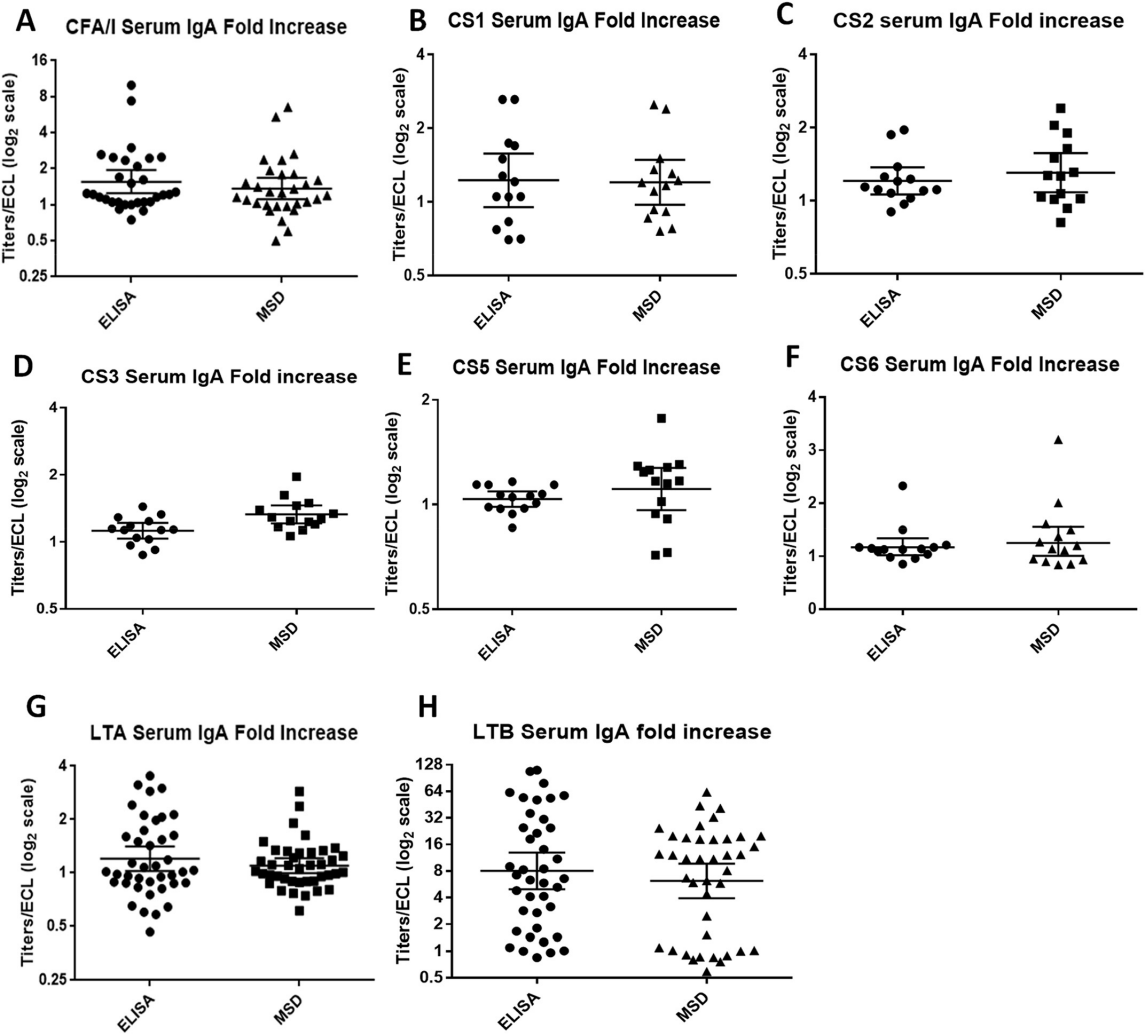
A–H Comparison between ELISA and ETEC multiplex ECL (MSD) for IgA with serum samples.

**Fig. 4:**
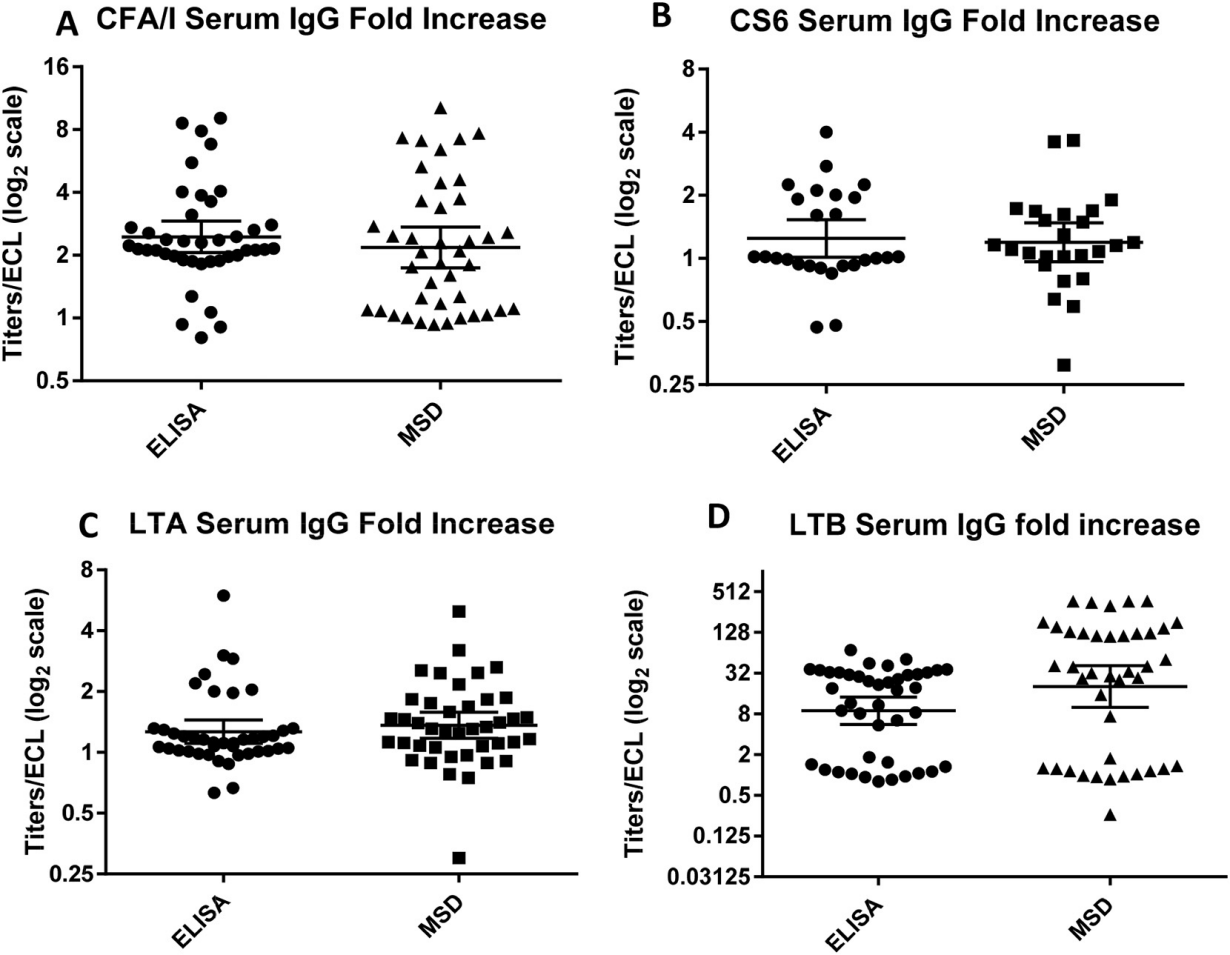
A–D Comparison between ELISA and ETEC multiplex ECL (MSD) for IgG with serum samples.

**Fig. 5:**
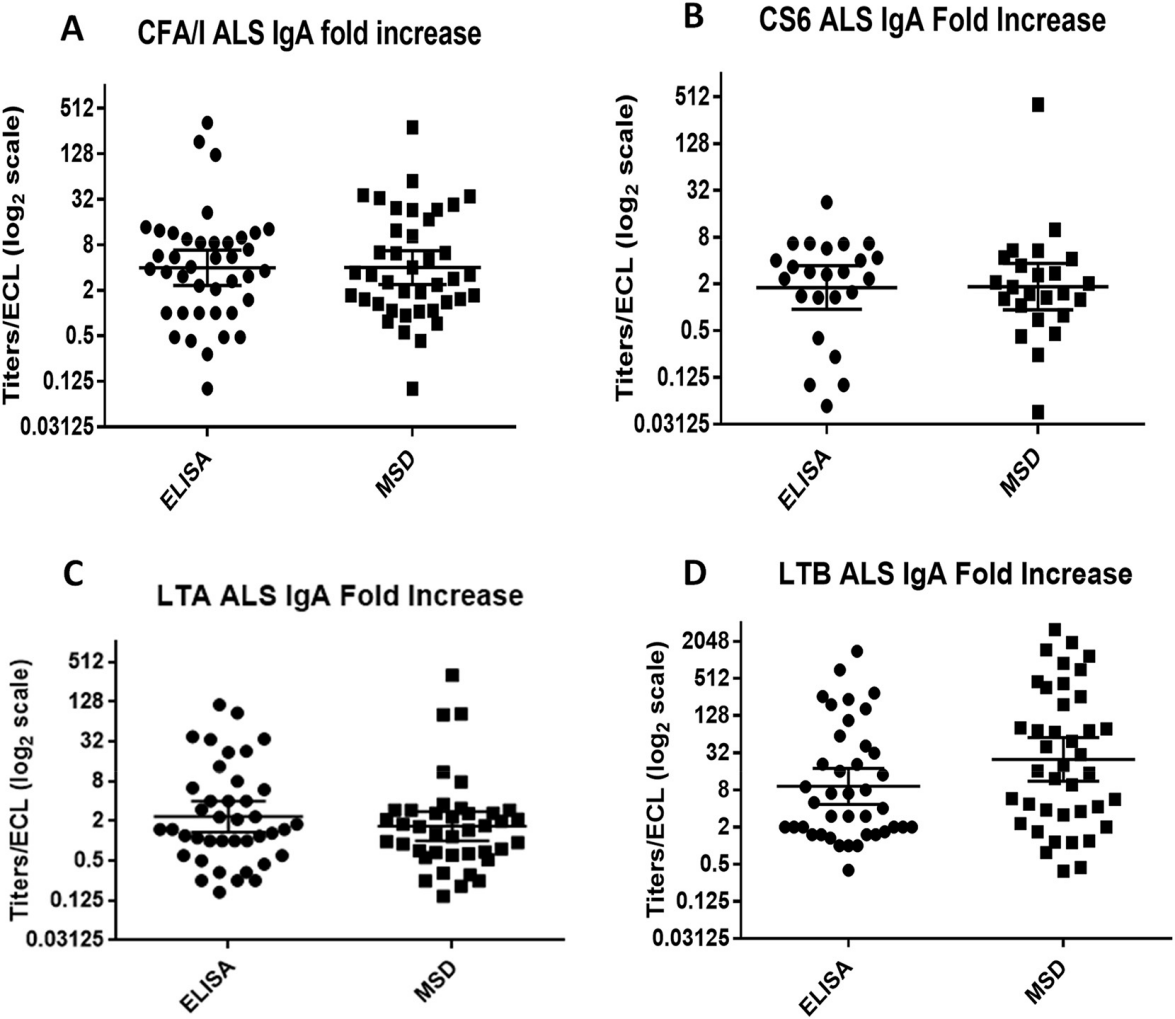
A–D Comparison between ELISA and ETEC multiplex ECL (MSD) for IgA with ALS samples.

To compare the multiplex ECL immunoassay with the conventional ELISA, serum samples were tested for IgA by both methods in parallel for 8 antigens. The samples included were serum samples collected before the first dose of ACE527 vaccine (baseline) and 7 days after the second dose of the vaccine (35 days after the first dose) along with 7 and 28 days after challenge with ETEC H10407 for both vaccinated and placebo volunteers. For analysis we used the data from the challenge phase only for CFA/I, LTA and LTB since these are the antigens present in the ETEC H10407 strain which was used to challenge the volunteers. The dilution of serum samples used was 1:500 as per the optimization study. The fold increase of the conventional ELISA titers and ECL signals from the baseline to after vaccination and after challenge were calculated, and plotted in the graph. The antibody titers of the 8 antigens from ELISA were compared with the ECL signals (Fig. 3 A–H). Student *t*-test analysis (*p* < .05) of the results obtained from the conventional ELISA vs the multiplex ECL immunoassay did not show any significant difference in the same set of samples tested by the two methods except for CS3 (*p* = .0087). For CS3, the fold increases from baseline were minimal in both of the assays, and the difference of GMT between the responses in the two assays was only 0.21 (Fig. 3D). Since the performance of serum IgA was satisfactory for all the 8 antigens tested, a second set of plates were coated with limited number of antigens (CFA/I, CS6, LTA and LTB) per well to test the performance of ETEC multiplex ECL in comparison with conventional ELISA for serum IgG and ALS IgA. We chose these antigens depending on the prevalence and importance of these antigens in vaccine development. The dilution of samples used for serum IgG was 1:500 and for ALS IgA was 1:3. The fold increase from the baseline was calculated and plotted in the graph for both ELISA and multiplex ECL (Fig. 4, Fig. 5). Similar to serum IgA, the two assays for IgG with serum did not show any significant difference (Fig. 4 A–D) except for LTB IgG in serum (*p* = .0200) (Fig. 4D). However, the patterns of responses in the two assays were similar and the individuals who responded in one assay also responded in the other but the magnitudes of responses in the MSD was higher compared to ELISA (Fig. 4C). This is likely because ECL is more sensitive than ELISA as the maximum to minimum ratio for ECL was 1434.62 compared to 87.63 for ELISA. Both methods performed similarly in distinguishing responses (4 fold or more increase from baseline) in ALS samples from individuals orally immunized with ACE527 vaccine or challenged with ETEC H10407 (Fig. 5 A–C). The responses for all the antigens tested were not significantly different in the two assays using ALS.

## 4 Discussion

The purpose of this study was to evaluate the feasibility of developing a multiplex ELISA for assessing the frequency, magnitude and breadth of serum and mucosal responses to ETEC CFs and toxin antigens using the MSD platform. As a proof of concept we developed an 8-plex assay with multiple CFs and LT toxin antigens. We optimized the coating and sample concentrations along with assay conditions for serum and ALS samples first in singleplex followed by multiplex format. To compare the ETEC multiplex MSD with ELISA, we carried out assays using the same antigens with the two immunoassay platforms using a common sample set, allowing an objective analysis of the relative sensitivities of the underlying technologies. The MSD platform achieved excellent correlations with ELISA for the antigens tested, consistently detecting antibody levels in the samples. Overall, the MSD technology appears to be more sensitive than ELISA. In addition, the excellent robustness of MSD, with high inter-assay precision at low analyte levels, resulted in comparable functional sensitivities for both platforms.

Current ETEC vaccine candidates are composed of the LT toxin subunit along with multiple CFs. In the absence of a defined correlates of protection from ETEC disease it is imperative to evaluate immune responses to all the antigens associated with the candidate ETEC vaccine, but this is difficult to achieve using traditional ELISA. Alternatively, the ETEC multiplex MSD is a high-throughput, dynamic, multi-array system which allows for the simultaneous measurement of over 10 antigen-specific antibodies from only 25–30 μl of highly diluted serum, compared to at least 1000 μl serum required to measure an equivalent number of antibodies by conventional ELISA. In addition, with this multiplex platform the amount of antigens (1–2 μl in MSD multiplex compared to ~100 μl per well in conventional ELISA), time for performing the assay, and human labor would also be immensely reduced. Therefore, the ETEC multiplex MSD that we developed could serve as a fundamental platform in evaluating performances of candidate ETEC vaccines. Since, using MSD multiplex platform a wider range of responses could be detected, this platform may perform better than ELISA in measuring immune responses to vaccines in children with pre-existing antibodies to ETEC in endemic settings.

This ETEC multiplex ECL can also be used as a tool for serosurveillance of the age/or regional distribution and prevalence of exposure to ETEC CFs in the endemic countries. Since the efficacy of the current candidate ETEC vaccines may depend on the protective immune responses to multiple ETEC CFs it would be crucial to know the circulating CF types in an area before vaccine is introduced. Pre-existing antibody levels against pathogens and toxins are of research interest, as they may positively correlate to eventual disease outcome. Despite conflicting results, it is evident that there is a growing trend in serological studies to establish the correlation between antibody levels and the disease outcome (Trauer et al., 2013), (Iyer et al., 2018), (Broderick et al., 2018).

The MSD platform is a technology well suited to routine laboratory operation. The reader has no requirement for regular maintenance or reagents. Data acquisition is rapid (plate read time similar to a conventional ELISA plate reader), and the software is user friendly. We and others have used this technique for determining human cytokine panel (Chakraborty et al., 2016), (Janot-Sardet et al., 2010). Multiplex MSD has been used for serum antibody levels eg., for the determination and detection of serum antibodies to Brucella strains (Thompson et al., 2009), quantification of human serum IgG against 10 *Staphylococcus aureus* toxins (Adhikari et al., 2016) and anti-pneumococcal antibodies in human serum which has been reported as highly sensitive and specific for the tested antigens (Marchese et al., 2009; Goldblatt et al., 2011). The MSD platform was also used for measuring anti-drug antibodies in serum samples in a phase III randomized, multinational trial of Motavizumab for prophylaxis of Respiratory Syncytial Virus in high-risk children (Carbonell-Estrany et al., 2010). The MSD plates are also available in different configurations, which could be useful for specific applications; for example, a 4-plex 384-well plate could be useful for disease outbreak testing.

There are some disadvantages to the MSD platform, such as relatively high reader and plate cost (compared to existing ELISA readers and plates) combined with specialist printing requirements of multiplex plates. However, because of the multiplex format the cost per sample for MSD is comparable to ELISA. The antigens we have used in ETEC ECL multiplex are not commercially available which may be a limiting factor. There are ongoing approaches from PATH, Department of Defense, Walter Read Army Institute of Research (WRAIR), Bill & Melinda Gates Foundation and National Institute of Health (NIH) to make these antigens available through a common source. Some of these antigens are available from the BEI resources repository at NIH (https://www.niaid.nih.gov/research/bei-resources-repository) which are from the same sources as used in this study.

In conclusion, the ETEC multiplex ECL assay provides a sensitive and specific technique for measuring antibodies, with the advantage of providing rapid results using small amounts of samples and antigens. This assay appears to be an acceptable alternative to the conventional ELISA for evaluating immune responses to ETEC antigens in phase III vaccine trials and disease surveillance studies. We are further evaluating the ETEC multiplex ECL assay to measure ETEC antigen specific immune responses from dried blood spots which would overcome the challenges involved in venous blood draws specifically from children, downstream specimen storage and transport methods in epidemiology studies in the endemic countries.

Funding

This work was supported by a grant from PATH, Washington, DC, USA. We thank all the volunteers in the ACE527 vaccine study. We acknowledge Barbara DeNearing, Fatema Mawanda, Joe Gomes for their contribution in the ACE527 vaccine trial. We thank Dr. AL Bourgeois and Dr. Richard Walker at PATH for their suggestions.
